# Serum selenium and vitamin E concentrations in indigenous Korean calves with neonatal weak calf syndrome

**DOI:** 10.1186/s13620-025-00326-y

**Published:** 2025-12-04

**Authors:** Youngwoo Jung, Byoungsoo Kim, Ji-Yeong Ku, Youngjun Kim, Kwang-Man Park, Jonghun Baek, Ji-Seon Yoon, DoHyeon Yu, John F. Mee, Jinho Park

**Affiliations:** 1 Department of Veterinary Internal Medicine, College of Veterinary Medicine, Jeonbuk National University, Iksan, Republic of Korea; 2 Institute of Animal Medicine, College of Veterinary Medicine, Gyeongsang National University, Jinju, Republic of Korea; 3 Department of Animal and Bioscience Research, Teagasc, Moorepark Research Centre, Fermoy, Co. Cork Ireland

**Keywords:** Colostrum, Indigenous Korean (Hanwoo) calf, Neonatal weak calf syndrome (NWCS), Selenium, Vitamin E

## Abstract

Neonatal weak calf syndrome (NWCS) is characterized by reduced vigour in neonatal calves, leading to difficulties in standing and suckling. This study aimed to evaluate whether serum selenium and Vitamin E (VE) concentrations were associated with NWCS. The study included 29 indigenous Korean (Hanwoo) beef calves: 10 healthy and 19 weak (14 surviving, 5 non-surviving), based on standing and suckling ability. These calves were recruited from 19 farms. Healthy calves suckled an adequate volume of colostrum while weak calves (who suckled inadequately) were fed 1–2 L of frozen colostrum or colostrum replacer; all within 2 h of birth. One blood sample was collected from each calf at least 4 h after last feeding (within 18 h after birth), and serum selenium and VE concentrations were analysed. The results showed no significant difference in serum selenium concentrations between healthy and weak calves but, VE concentrations were significantly lower (and deficient) in weak calves. Among the weak calves, non-survivors also showed numerically lower levels than survivors. These findings suggest an association between calf blood VE concentrations and NWCS but not with calf blood selenium concentrations. It is hypothesised that the NWCS was caused by foetal VE deficiency as despite additional colostrum feeding weak calves (NWCS) still had significantly lower postcolostral serum VE concentrations than healthy calves. As VE is involved in muscle function, immunity, and oxidative stress regulation, ensuring adequate maternal VE supplementation and timely colostrum intake may help reduce both the impact of VE deficiency on NWCS and the impact of NWCS on VE status in calves with low maternal–foetal VE reserves where prompt colostrum feeding is not practised, as is often the case with beef calves.

## Introduction

Weak calf syndrome (WCS) has been variously defined as occurring during the perinatal or neonatal period. For clarity, we have introduced two new acronyms: perinatal WCS (PWCS) and neonatal WCS (NWCS). The PWCS most commonly occurs in dairy heifers and is associated with bradytocia, stillbirth or death within minutes of birth, and premature placental expulsion [1–3]. Neonatal WCS, also referred to as ‘dummy calf’ or ‘fading calf’, is a broad term used to describe calves with reduced vigour after birth, characterized by delayed or absent spontaneous standing and/or a weak or absent suckling response. Affected calves may die several days after birth [4–6]. There are multiple causes of WCS including dystocia, as well as environmental, genetic, infectious, and nutritional factors [5–10]. For example, calves born following dystocia often experience respiratory and metabolic acidosis, and hypothermia due to failure of physiological adaptation. Without appropriate intervention, these calves are at a high risk of NWCS or dying within the first few days of life [11].

Calves with reduced vigour, manifested as impaired standing or suckling, are more likely to experience delayed, insufficient, or poorly absorbed colostrum intake, regardless of the underlying cause [12]. Colostrum serves as a primary source of immunoglobulins, essential vitamins, and minerals, such as selenium and vitamin E (VE) [13].

Selenium and VE have complementary roles in oxidative defence and immune regulation in ruminants. Selenium is a key component of glutathione peroxidase (GSH-Px), which protects cells from oxidative damage by reducing hydrogen peroxide and lipid hydroperoxides [14, 15]. Vitamin E, a lipid-soluble antioxidant composed of tocopherols and tocotrienols, prevents lipid peroxidation in cell membranes, thereby preserving membrane integrity [14, 16]. Selenium and VE also modulate immune responses, particularly under oxidative stress (OS). Neonatal calves exhibit elevated OS but possess immature antioxidant systems, making them especially vulnerable to oxidative damage in early life [17].

Deficiencies in selenium and/or VE can lead to significant physiological disturbances in neonatal calves. Selenium deficiency is known to cause nutritional muscular dystrophy, or white muscle disease (WMD), which results from degenerative lesions in skeletal and cardiac muscle, leading to weakness, impaired mobility, and in severe cases, death [14]. Selenium deficiency has also been associated with perinatal mortality [18, 19]. However, although maternal selenium supplementation may improve both maternal and foetal selenium status, it may not reduce perinatal mortality if selenium deficiency is not the primary underlying cause [20]. Vitamin E deficiency has been associated with oxidative damage, potentially leading to muscular degeneration and impaired immune function. These effects may contribute to perinatal mortality [21] and increased susceptibility to infectious diseases, both of which can cause neonatal weakness during the periparturient period, independently of selenium deficiency [14, 22]. When both deficiencies occur concurrently, the disruption of their synergistic antioxidant roles exacerbates OS, further compromising neonatal viability [16]. Therefore, selenium and VE are commonly administered parentally as a preventive measure to newborn calves in some regions, such as North America.

Thus, poor neonatal viability or WCS may result from selenium and/or VE deficiency, due to low foetal selenium and VE status and/or inadequate or delayed intake of colostrum containing these nutrients. However, WCS itself may also lead to inadequate or delayed colostrum intake, contributing to low neonatal selenium and VE status, suggesting a bidirectional relationship.

A previous study investigating physiological alterations and mortality predictors in weak calves identified predictive blood parameters such as pCO₂, pH, and ALP levels [23]. To extend our understanding of the NWCS, the hypothesis tested in this study was that, given the associations between micronutrients and WCS, calf blood selenium and VE concentrations would be lower in weak than in healthy calves after birth.

## Materials and methods

### Calves

This prospective case series study was conducted between December 2023 and April 2024 in Yeonggwang, located in the southwestern region of Korea. The average ambient temperatures during the study period ranged from 2.0 °C to 15.8 °C. Twenty-nine indigenous Korean beef (Hanwoo) calves born at full term of at least 260 days of gestation were recruited to this study from 19 farms. No apparent infectious disease outbreaks occurred during the study period and in the preceding year. When a farmer observed that a calf was weak after birth, characterized by an inability to stand and/or a poor or absent suck reflex, they administered colostrum or colostrum replacer as outlined below and contacted their veterinarian (B. Kim). Upon arrival, the veterinarian conducted a clinical assessment of each affected calf and collected blood samples from both the affected and age-matched healthy control calves.

### Calving and feeding management

The dams were housed in individual pens on the farm and monitored during late pregnancy. They were fed a combination of grass silage (approximately 14–18 kg/cow/day) and concentrate feeds (approximately 2–4 kg/cow/day), without any additional supplementation of selenium or VE. Calves were delivered naturally except for two that required mild assistance due to delayed parturition. No mechanical traction or surgical intervention was involved.

After birth, initial calf observation and care were performed by the farmers, and colostrum feeding was initiated immediately thereafter. If the calf was healthy, it was either left to suckle colostrum directly from the dam, with intake confirmed by observation of active suckling behaviour and evidence of udder emptying, or was separated and fed 1–2 L of frozen colostrum or 300–500 g of colostrum replacer (CR) using either a bottle or an oesophageal tube feeder, depending on individual farm protocol. If the calf was weak and unable to suckle effectively, it was separated and fed the same volume of frozen colostrum or CR using an oesophageal tube feeder. Multiple commercial CR products were used. Following this initial feeding, all calves were deliberately kept in individual pens and were fed CR twice daily by farmers throughout the 7-day study period.

### Calf assessment and classification

**Fig. 1:**
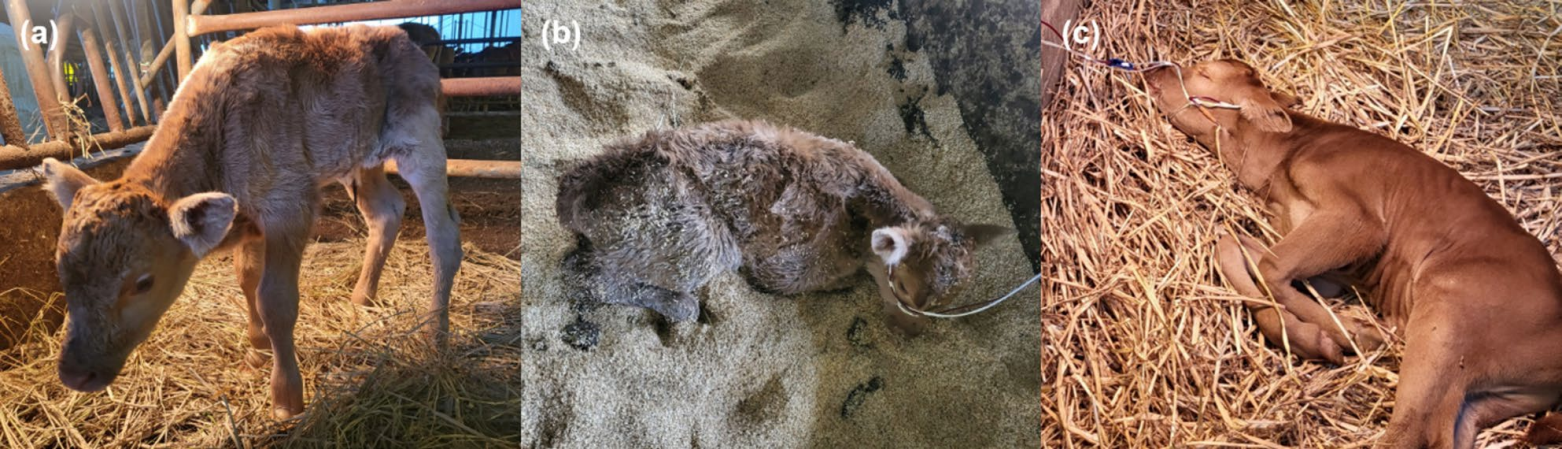
Hanwoo weak calves included in the study. **a** A weak calf standing which exhibited a weak suckling reflex. **b** A weak calf unable to maintain a standing posture. **c** A weak calf with lethargy, unable to maintain a sternal recumbency position

Calves were classified by the veterinarian as healthy (*n* = 10) if they could stand unassisted and/or exhibited a strong suckling reflex. Weak calves (*n* = 19) were defined as those that failed to attempt standing, were unable to maintain an upright position on all four limbs, or exhibited a weak or absent suckling reflex when a finger was inserted into the oral cavity (Fig. 1). All weak calves originated from different farms. Healthy calves from four of these farms were included as controls, as these were the only farms where healthy calves were present at the time of sampling.

Following classification, blood collection was conducted at least 4 h after the last colostrum or CR feeding. The mean ± SD age of calves at blood sampling was 17.7 ± 9.9 h (range: 4–32 h). Therapeutic interventions were initiated after blood collection. The calves were monitored daily by a veterinarian for one week to assess vigour and survival. Weak calves were further classified into surviving (*n* = 14) and non-surviving (*n* = 5) groups based on whether they survived through the first 7 days of life.

### Blood collection and analysis

To analyse serum VE and selenium concentrations, one blood sample was collected anaerobically from the jugular vein of each calf into 5 mL serum-separating tubes (Vacuette serum tube; Greiner Bio-One, Kremsmünster, Austria). After blood collection, the tubes were left at ambient temperature and then transported to the laboratory under refrigeration within 1 h. At the laboratory, the tubes were left to stand for at least 1 h, and the serum was separated by centrifugation at 3000 × *g* for 10 min. The separated serum was stored in a freezer at − 24 °C and tested within a week. Frozen serum samples were thawed at room temperature for 30 min before analysis. Serum selenium concentrations were measured using inductively coupled plasma mass spectrometry (ICP-MS) with an Agilent ICP-MS system (Agilent Technologies, Santa Clara, CA, USA). Serum VE (D-α-tocopherol) was analysed by high-performance liquid chromatography (HPLC) using an Agilent HPLC system (Agilent Technologies, Santa Clara, CA, USA) and a Vitamin A/E Reagent Kit (Chromsystems, Gräfelfing, Germany).

### Statistical analysis

Data were analysed using the SPSS statistical software package (SPSS 21.0; IBM SPSS Statistics, Armonk, NY, USA) and GraphPad Prism 8 software (GraphPad Software Inc., San Diego, CA, USA). The normality of the data was assessed using the Kolmogorov–Smirnov and Shapiro–Wilk tests. As most data were not normally distributed, results were presented as the median and interquartile range (IQR). The Mann–Whitney U test was used to compare the measured parameters between healthy and weak calves, as well as between surviving and non-surviving weak calves. Statistical significance was set at *p* < 0.05.

## Results

There was no significant difference in selenium concentrations between healthy and weak calves (*p* = 0.195). The median (IQR) selenium concentration was 72.35 (58.53–100.43) µg/L in healthy calves and 59.55 (21.00–88.53) µg/L in weak calves.

In contrast, the VE (D-α-tocopherol) concentrations were lower in weak calves than in healthy calves (*p* < 0.01). The median (IQR) VE concentration was 0.94 (0.82–1.28) µg/mL in healthy calves and 0.37 (0.25–0.54) µg/mL in weak calves (Table 1).

**Table 1:** Median (IQR) serum selenium and vitamin E concentrations in healthy and weak Hanwoo calves within 32 h of birth and at least 4 h after last colostrum or colostrum replacer feeding

Micronutrient	Healthy calves (*n* = 10)	Weak calves (*n* = 19)	*p*-value
Selenium (µg/L)	72.35 (58.53–100.43)	59.55 (21.00–88.53)	0.195
Vitamin E (D-α-tocopherol) (µg/mL)	0.94 (0.82–1.28)	0.37 (0.25–0.54)	< 0.01

**Fig. 2:**
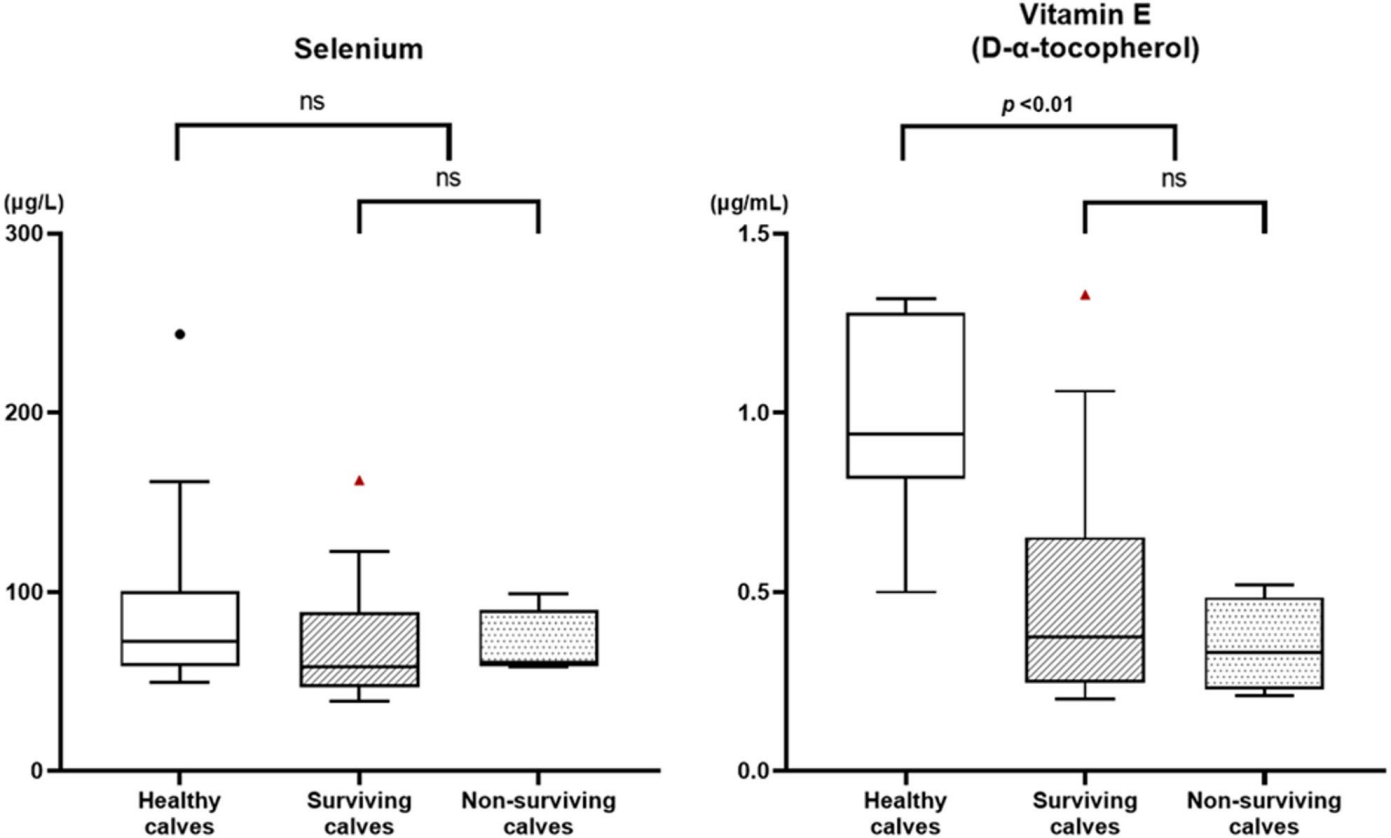
Serum selenium and vitamin E concentrations in healthy, surviving weak, and non-surviving weak calves. Data are presented as box-and-whisker plots (Tukey method), showing the median, interquartile range (IQR), and values within 1.5 × IQR. Outliers are indicated as black circles (healthy calves) and red triangles (surviving weak calves). Statistical comparisons were performed using the Mann–Whitney U test. ‘ns’ indicates a non-significant difference (p > 0.05)

Weak calves were divided into surviving and non-surviving subgroups for comparison. There was no significant difference in serum selenium or VE concentrations between the two groups. The median (IQR) selenium concentration was 58.25 (46.45–88.53) µg/L in surviving weak calves and 60.90 (58.33–89.80) µg/L in non-surviving weak calves (*p* = 0.395). The median (IQR) VE concentration was 0.38 (0.25–0.65) µg/mL in surviving weak calves and 0.33 (0.23–0.49) µg/mL in non-surviving weak calves (*p* = 0.670) (Fig. 2).

## Discussion

In this study, we analysed the serum selenium and VE concentrations in Hanwoo calves with NWCS to evaluate their association with the presentation of the condition. Serum VE concentrations were lower in weak calves than in healthy calves. In contrast, serum selenium concentrations did not differ among healthy, weak surviving, and weak non-surviving calves. Thus, our findings only partially support our hypothesis, suggesting an association between low VE status and NWCS, but not between selenium status and NWCS.

To interpret these findings, it is important to evaluate whether the measured concentrations were within normal or deficient ranges. When assessing serum analytes in neonates, adult reference ranges are often inappropriate due to physiological differences. Reported reference ranges for selenium include 65–140 ng/mL for adults and 20–70 ng/mL for neonates [24]. Additionally, selenium concentrations of 0.2–0.7 µmol/L in calves aged 1–6 days and 0.5–3.8 µmol/L in cows have been documented [25], corresponding to approximately 15.8–55.3 µg/L and 39.5–300.0 µg/L, respectively.

Based on these ranges, serum selenium concentrations in the present study fell within or above the expected values for neonatal calves. Therefore, the absence of an association between selenium status and NWCS is unsurprising. Similar findings have been reported, where selenium supplementation marginally increased calf selenium status but did not reduce the incidence of WCS [3]. Retrospective analyses have also suggested that although selenium status can be important for calf health, it does not consistently explain weakness at birth [18]. In contrast, low whole-blood selenium concentrations have been linked to stillbirths, perinatal mortality, and weakness in beef calves [19]. This discrepancy may suggest that selenium deficiency acts as a threshold factor. Vulnerability increases markedly once concentrations fall below a critical level, whereas above this threshold, additional selenium may have limited impact on neonatal viability. It is also possible that adverse outcomes previously attributed to selenium deficiency reflect combined deficiencies of both selenium and VE, rather than selenium alone. From this perspective, our results imply that while selenium levels in Hanwoo calves were likely sufficient, prenatal VE deficiency may have played a more decisive role in the onset of NWCS. Furthermore, a recent report found no clear association between serum selenium concentrations and weak calf outcomes, which appears to reinforce our findings [26].

In contrast, VE concentrations in the NWCS group were deficient when compared to the neonatal reference range (0.7–3.5 µg/mL) [27], and lower than in healthy calves. These findings suggest that VE deficiency may be associated with NWCS, whereas selenium status is unlikely to be a major contributing factor, albeit based on serum, not tissue analyses. Consistent with prior reports, lower neonatal α-tocopherol has been associated with increased early mortality, particularly when passive transfer fails [28].

To explain these divergent findings, a review of perinatal selenium and VE dynamics is apposite. Selenium readily crosses the bovine placenta and is preferentially partitioned to the foetus, often resulting in higher foetal blood selenium concentrations than those in the dams [29]. Moreover, maternal selenium supplementation during late pregnancy increases colostral selenium concentration [30]. Thus, bovine neonates have two sources of selenium, directly from the dam in utero and indirectly from the dam via colostrum. In the present study, healthy calves were able to suckle naturally, whereas weak calves required artificial feeding. The similar and normal post-colostral selenium concentrations between groups suggest that both routes of colostrum delivery were sufficient to meet neonatal selenium needs and/or that foetal selenium stores were already adequate. This likely explains the lack of differences in serum selenium levels between healthy and weak calves.

As a result, neonatal calves depend almost entirely on colostrum to establish circulatory VE concentrations [31]. Colostrum contains much higher concentrations of VE than milk, and maternal VE supplementation during late pregnancy enhances this content [14, 16]. But if calves are weak after birth with conditions such as WCS, this potentially reduces both colostrum intake and absorption of VE, in the absence of prompt colostrum feeding. In this study, both healthy and weak calves received colostrum within hours of birth, thereby reducing disparities in VE intake. However, weak calves still showed lower VE concentrations, suggesting a pre-existing foetal VE deficiency. This may indicate a feed-forward loop in which foetal VE deficiency contributes to weakness at birth, and that weakness further limits VE uptake.

Blood samples were collected at least four hours after the final colostrum feeding. Previous studies have demonstrated increases in serum α-tocopherol and plasma selenium within 12 and 24 h, respectively, following colostrum ingestion [32, 33]. Some absorption of selenium and VE may have occurred by the time of sampling. However, the consistently low serum VE concentrations in most weak calves and some healthy calves suggest that a prenatal deficiency was already established. It remains unclear whether there were differences in micronutrient intake between calves that suckled naturally and those that were fed colostrum or CR.

Given these findings, the role of VE deficiency in WCS needs to be explained. Foetal deficiency of VE, together with selenium deficiency, has been associated with congenital myopathy and cardiomyopathy which may lead to abortion, congenital WMD and WCS [34, 35].

Our findings suggest that VE deficiency may be associated with NWCS. However, because samples were not collected prior to colostrum ingestion or the onset of clinical signs, causation cannot be confirmed. The relationship between VE deficiency and NWCS may also be bidirectional.

This study has certain limitations. As an observational study, it cannot establish causality between micronutrient status and NWCS. The most important limitation is that all blood samples were collected only after colostrum or CR feeding. Therefore, it is not possible to determine whether the lower serum VE concentrations observed in weak calves preceded the development of NWCS, resulted from impaired colostrum intake or absorption due to NWCS, or reflected a bidirectional relationship between the two. To draw stronger conclusions, future studies should assess pre-colostral serum levels and colostrum composition. The relatively small sample size, particularly for the non-surviving group, may have limited the ability to detect significant associations. Additionally, the study was restricted to Hanwoo calves from a single region, which may limit generalizability. Whether genotype interacts with WCS susceptibility remains unknown. The concentrations of selenium and VE in both colostrum and CR were not measured in this study. Therefore, it remains unclear whether the low serum VE concentrations in weak calves resulted from insufficient supply, impaired absorption, or pre-existing foetal deficiency. Maternal micronutrient status and the causes of death in non-surviving calves were also not determined. Moreover, only a single post-partum sampling time point was analysed, and the study focused exclusively on selenium and VE. As a result, temporal changes in micronutrient status and their relationship with other biochemical or haematological parameters could not be evaluated.

Despite the lack of a statistically significant association with mortality, the potential influence of VE status on survival cannot be excluded. Future studies should incorporate multiple sampling points and a broader range of biomarkers, alongside larger, multi-regional cohorts, to clarify these associations. Given the potential interactions among genotype, maternal nutrition, and perinatal care, further research using standardized protocols in diverse environments is essential to develop a unifying understanding of NWCS pathogenesis.

## Data Availability

The data supporting the findings of this study are available from the corresponding author upon reasonable request.

## Abbreviations

CR: Colostrum replacer
GSH-Px: Glutathione peroxidase
HPLC: High performance liquid chromatography
ICP-MS: Inductively coupled plasma mass spectrometry
IQR: Interquartile range
NWCS: Neonatal weak calf syndrome
OS: Oxidative stress
PWCS: Perinatal weak calf syndrome
SD: Standard deviation
VE: Vitamin E
WCS: Weak calf syndrome
WMD: White muscle disease
